# Dermoscopic Features of Tinea Capitis in Korean Patients: Correlation With Clinical Subtypes, Age Groups and Causative Dermatophytes

**DOI:** 10.1111/myc.70184

**Published:** 2026-05-18

**Authors:** Thi Quynh Trang Tran, Gwangil Kim, Hyun‐Ji Ryu, Su‐Kyung Park, Kyung‐Hwa Nam, Seok‐Kweon Yun, HyoHyun Yoo, Jin Park

**Affiliations:** ^1^ Department of Dermatology, College of Medicine Jeonbuk National University Jeonju Republic of Korea; ^2^ Department of Dermatology, University of Medicine and Pharmacy Hue University Hue Vietnam; ^3^ Research Institute of Clinical Medicine of Jeonbuk National University‐Biomedical Research Institute of Jeonbuk National University Hospital Jeonju Republic of Korea; ^4^ Department of Medical Education, Medical School Jeonbuk National University Jeonju Republic of Korea

**Keywords:** dermatomycoses, dermoscopy, *microsporum*, tinea capitis, *trichophyton*

## Abstract

**Background:**

Tinea capitis (TC) presents with diverse clinical manifestations that may complicate diagnosis. Dermoscopy is a useful adjunctive tool, but data correlating dermoscopic findings with clinical and mycological characteristics in Korean patients remain limited.

**Objective:**

To characterize dermoscopic features of TC in Korean patients and evaluate their associations with clinical subtypes, age distributions and causative dermatophytes.

**Methods:**

We retrospectively analysed 71 patients with mycologically confirmed TC diagnosed between 2011 and 2025. Dermoscopic patterns were compared across clinical subtypes, fungal species and age groups. Representative dystrophic hairs were further examined using super‐resolution confocal laser scanning microscopy.

**Results:**

The cohort demonstrated a bimodal age distribution, with peaks in children (≤ 10 years) and elderly adults (≥ 61 years). *Microsporum canis* was the predominant pathogen (71.8%). The most frequent dermoscopic findings were scaling (83.1%), black dots (78.9%), follicular hyperkeratosis (78.9%) and broken hairs (78.9%). Empty follicles were significantly associated with *Trichophyton* infections (*p* = 0.001), whereas zigzag hairs, Morse code–like hairs, bent hairs and scaling were significantly more frequent in *Microsporum* infections (*p* < 0.05). Comma hairs were commonly observed in diffuse pustular and black dot subtypes. Children more often exhibited Morse code–like hairs, whereas elderly patients showed higher rates of empty follicles and perifollicular erythema. Confocal microscopy confirmed distinct structural shaft damage corresponding to characteristic dermoscopic patterns.

**Conclusion:**

Dermoscopy provides valuable diagnostic and aetiological clues in TC, enabling differentiation between clinical subtypes and prediction of causative fungal species. These findings are especially relevant in adult‐predominant, *Microsporum*‐endemic settings and support dermoscopy as an important adjunct to routine clinical evaluation.

## 1 Introduction

Tinea capitis (TC) is a superficial dermatophyte infection that involves the hair follicles and shafts of the scalp, most commonly caused by *Trichophyton* or *Microsporum* species [1]. It predominantly affects school‐age children but may also occur in adults, particularly in the elderly, with recent epidemiological studies reporting a gradual shift towards adult‐onset cases [2, 3]. Transmission occurs through direct contact with infected humans or animals, as well as indirectly via contaminated fomites. Zoophilic dermatophytes are known to provoke more pronounced inflammatory responses than anthropophilic species [4].

Clinically, TC presents with a broad spectrum of manifestations ranging from non‐inflammatory alopecic patches to highly inflammatory forms such as kerion celsi. It is generally categorized into inflammatory and non‐inflammatory forms, encompassing five clinical subtypes: grey patch, black dot, seborrhoeic dermatitis‐like diffuse scaling, diffuse pustular type and kerion. The heterogeneity of clinical presentations frequently leads to delayed diagnosis or misdiagnosis, particularly in adults [5].

Mycological examination, including potassium hydroxide (KOH) microscopy and fungal culture, remains essential for confirming TC [5]. However, these methods require time and technical expertise, and fungal cultures may take several weeks to yield results. Dermoscopy has emerged as a rapid, non‐invasive adjunctive diagnostic tool that allows visualization of characteristic hair shaft and follicular abnormalities not detectable by the naked eye [6, 7, 8, 9]. Previous studies have identified several dermoscopic features suggestive of TC, such as comma hairs, corkscrew hairs, Morse code–like hairs and black dots, which may facilitate differentiation from other causes of patchy alopecia [6, 8, 10, 11].

Although dermoscopic features of TC have been described in multiple prospective and retrospective studies, most reports have focused on paediatric populations or mixed ethnic cohorts. Data on Korean patients remain limited, and the association between dermoscopic findings and demographic factors, clinical subtypes and causative fungal species has not been systematically evaluated. Therefore, this study aimed to characterize the dermoscopic features of TC in a Korean population and to analyse their correlations with demographic, clinical and mycological characteristics.

## 2 Patients and Methods

We conducted a single‐centre retrospective study of patients diagnosed with tinea capitis at Jeonbuk National University Hospital between 2011 and 2025. A total of 71 patients with mycologically confirmed TC, based on positive KOH examination and/or fungal culture, were included.

Medical records were reviewed to collect demographic and clinical information, including age, sex, site of scalp involvement, number of lesions, hair curl type (straight, wavy or curly), clinical subtype and suspected source of infection. TC was clinically classified into five subtypes: inflammatory (kerion celsi and diffuse pustular type) and non‐inflammatory (grey patch type, seborrhoeic dermatitis–like diffuse scaling type and black dot type). Diagnostic evaluations included Wood's lamp examination, KOH microscopy of scalp scales and hair shafts, and fungal culture. Polymerase chain reaction (PCR) targeting internal transcribed spacer regions was performed for fungal species identification. Patterns of hair invasion were categorized as ectothrix or endothrix based on microscopic or histopathological findings when available.

Dermoscopy was performed using polarized handheld dermoscopes (DermLite DL3 and DL5; 3Gen LLC, San Juan Capistrano, CA, USA) at 10× magnification. Images were captured using a digital camera system. Dermoscopic features were categorized into follicular, perifollicular, hair shaft, vascular and other scalp findings. Image assessment was conducted retrospectively based on archived dermoscopic photographs. Dermoscopic images were independently reviewed by two dermatologists with expertise in trichoscopy, who were blinded to the clinical and mycological data. Any discrepancies were resolved by consensus.

In representative cases, infected dystrophic hairs showing characteristic morphologic abnormalities were obtained by hair plucking. The collected hair shafts were examined using super‐resolution confocal laser scanning microscopy (LSM 880 with Airyscan; Carl Zeiss, Oberkochen, Germany) at the Center for University‐Wide Research Facilities (CURF), Jeonbuk National University. The specimens were mounted on glass slides without additional staining and examined to evaluate ultrastructural alterations of the hair shaft and fungal elements. High‐resolution images were acquired to correlate microscopic changes with dermoscopic features.

Statistical analyses were performed using IBM SPSS Statistics version 27.0. Categorical variables were compared using Pearson's chi‐squared test. When the assumptions for the chi‐squared test were not met (i.e., when more than 20% of expected cell counts were < 5), Fisher's exact test or the likelihood ratio chi‐squared test was applied, as appropriate. A two‐sided *p*‐value < 0.05 was considered statistically significant. For variables showing significant overall differences, post hoc pairwise comparisons were performed using the z‐test for independent proportions with the Bonferroni correction applied to adjust for multiple testing.

This study was approved by the Institutional Review Board of Jeonbuk National University Hospital (JBUH 2013‐09‐015) and conducted in accordance with the Declaration of Helsinki. Informed consent was obtained from all participants.

## 3 Results

### 3.1 Demographic and Clinical Characteristics

A total of 71 patients with mycologically confirmed tinea capitis were included in this study. The demographic and clinical characteristics are summarized in Table 1. The mean age of the patients was 41.06 years (range, 2–90 years). The age distribution showed a bimodal pattern, with peaks in children aged ≤ 10 years (45.1%) and older adults aged ≥ 61 years (45.1%). There was a female predominance, with a male‐to‐female ratio of 1:2.09. Regarding hair curl type, straight hair was most common (63.4%), followed by wavy (26.8%) and curly hair (9.8%).

**TABLE 1:** Epidemiological and clinical characteristics of patients with tinea capitis (*n* = 71).

Clinical information	Number of cases (%)
Mean age, years (range)	41.06 (2–90)
≤ 10	32 (45.1)
11–30	3 (4.2)
31–50	1 (1.4)
51–60	3 (4.2)
≥ 61	32 (45.1)
Sex (M:F = 1:2.09)	
Male	23 (32.4)
Female	48 (67.6)
Location	
Parietal	36 (50.7)
Occipital	30 (42.3)
Frontal	17 (23.9)
Vertex	23 (32.4)
Temporal	9 (12.7)
Hair curling	
Straight	45 (63.4)
Wavy	19 (26.8)
Curly	7 (9.8)
Number of lesions	
Single	22 (31.0)
Multiple	49 (69.0)
Clinical classification	
Non‐inflammatory type	
Grey patch type	26 (36.6)
Seborrhoeic dermatitis–like diffuse scaling type	11 (15.5)
Black dot type	5 (7.1)
Inflammatory type	
Kerion celsi	16 (22.5)
Diffuse pustular type	13 (18.3)

Lesions were most frequently located on the parietal scalp (50.7%), followed by the occipital (42.3%) and vertex (32.4%) regions, while temporal involvement was relatively uncommon (12.7%). Multiple alopecic lesions were observed in 69.0% of patients.

**FIGURE 1:**
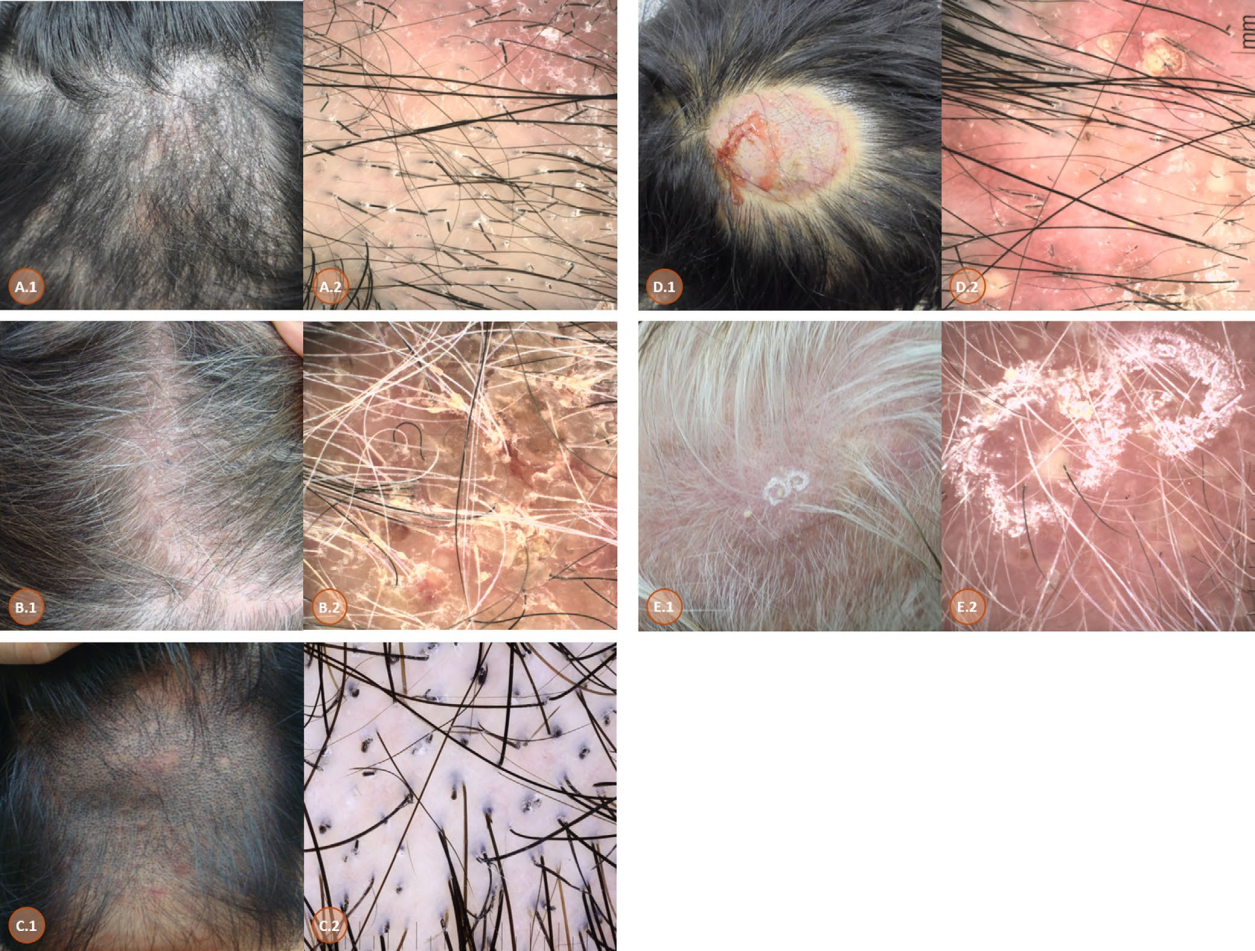
Dermoscopic findings of tinea capitis by clinical subtype and causative fungal species (A) Grey patch type caused by *Microsporum canis*, showing perifollicular scaling, Morse code–like hairs and bent hairs. (B) Seborrhoeic dermatitis‐like diffuse scaling type caused by *Trichophyton rubrum*, showing diffuse scales. (C) Black dot type caused by *Trichophyton tonsurans*, showing black dots, comma hairs and short broken hairs. (D) Kerion celsi caused by *Microsporum canis*, showing diffuse erythema, crusting, pustules, comma hairs and broken hairs. (E) Diffuse pustular type caused by *Microsporum canis*, showing diffuse erythema, black dots, perifollicular pustules, broken hairs and comma hairs.

Clinically, non‐inflammatory types were more common than inflammatory types, although the difference was not statistically significant. Among the five clinical subtypes, the grey patch type was the most prevalent (36.6%), followed by kerion celsi (22.5%), diffuse pustular type (18.3%), seborrhoeic dermatitis‐like diffuse scaling type (15.5%) and black dot type (7.1%) (Figure 1).

### 3.2 Mycological Characteristics

Mycological findings are detailed in Table 2. A suspected zoonotic source of infection was identified in 50.7% of cases with available data. Wood's lamp examination was positive in 49.3% of patients. Direct microscopic examination with potassium hydroxide (KOH) showed high positivity rates in both scalp scales (93.0%) and hair shafts (90.1%).

**TABLE 2:** Mycological characteristics of 71 patients with tinea capitis.

Mycological information	Number of cases (%)
Predicted source of infection (double answer possible)	
Animal (e.g., cat, dog, cow)	38 (50.7)
Other human contact (e.g., wrestler)	1 (1.4)
Not available	34 (47.9)
Wood light	
Positive	35 (49.3)
Negative	15 (21.1)
Not available	21 (29.6)
KOH examination (double result possible)	
Scalp positive	66 (93.0)
Hair positive	64 (90.1)
Morphologic classification	
Ectothrix	52 (73.2)
Endothrix	11 (15.5)
Not available	8 (11.3)
Fungal pathogen	
*Microsporum canis*	51 (71.8)
*Trichophyton rubrum*	6 (8.5)
*Trichophyton tonsurans*	3 (4.2)
*Trichophyton mentagrophytes*	2 (2.8)
*Trichophyton verrucosum*	1 (1.4)
No growth	5 (7.1)
Contaminated culture/unavailable identification	3 (4.2)

Regarding the pattern of fungal invasion, ectothrix parasitism was predominant (73.2%), whereas endothrix involvement was observed in 15.5% of cases. *Microsporum* species were the most common causative pathogens, accounting for 71.8% of cases, with *Microsporum canis* being the single most frequently isolated organism. *Trichophyton* species accounted for 16.9% of cases, with *Trichophyton rubrum* being the most common (8.5%). Fungal culture showed no growth or contamination in 11.3% of cases.

### 3.3 Dermoscopic Features According to Clinical Subtypes

Dermoscopic findings across the five clinical subtypes are summarized in Table 3 and Figure 1. Dermoscopic findings differed significantly across the five clinical subtypes of tinea capitis.

**TABLE 3:** Dermoscopic features of tinea capitis according to clinical subtype (*n* = 71).

Dermoscopic feature	Total (*n* = 71)	Non‐inflammatory subtypes			Inflammatory subtypes		*p*
		GP (*n* = 26)	SD (*n* = 11)	BD (*n* = 5)	K (*n* = 16)	DP (*n* = 13)	
Follicular opening							
Black dots	56 (78.9)	18 (69.2)	8 (72.7)	5 (100.0)	13 (81.3)	12 (92.3)	0.220
Fibrotic white dots	1 (1.4)	0 (0.0)	0 (0.0)	0 (0.0)	1 (6.3)	0 (0.0)	0.553
Follicular hyperkeratosis	56 (78.9)	18 (69.2)	9 (81.8)	5 (100.0)	13 (81.3)	11 (84.6)	0.394
Empty follicles	30 (42.3)	5 (19.2)^b^	8 (72.7)^a^	3 (60.0)^a,b^	8 (50.0)^a,b^	6 (46.2)^a,b^	0.021
Perifollicular							
Perifollicular erythema	31 (43.7)	5 (19.2)^a^	5 (45.5)^a,b^	2 (40.0)^a,b^	8 (50.0)^a,b^	11 (84.6)^b^	0.002
Perifollicular pustules/vesicles	30 (42.3)	3 (11.5)^a^	2 (18.2)^a^	0 (0.0)^a^	13 (81.3)^b^	12 (92.3)^b^	< 0.001
Perifollicular scale	54 (76.1)	22 (84.6)	9 (81.8)	4 (80.0)	8 (50.0)	11 (84.6)	0.133
Hair pattern							
Broken hair	56 (78.9)	21 (80.8)	8 (72.7)	5 (100.0)	12 (75.0)	10 (76.9)	0.586
Comma hair	42 (59.2)	14 (53.8)^a,b^	2 (18.2)^a^	4 (80.0)^a,b^	11 (68.8)^a,b^	11 (84.6)^b^	0.008
Corkscrew hair	35 (49.3)	12 (46.2)	4 (36.4)	4 (80.0)	7 (43.8)	8 (61.5)	0.448
Zigzag hair	34 (47.9)	14 (53.8)	3 (27.3)	1 (20.0)	7 (43.8)	9 (69.2)	0.179
Morse code–like hair	38 (53.5)	18 (69.2)	3 (27.3)	2 (40.0)	7 (43.8)	8 (61.5)	0.139
Bent hair	46 (64.8)	21 (80.8)	6 (54.5)	3 (60.0)	8 (50.0)	8 (61.5)	0.260
Vessels							
Arborizing vessels	31 (43.7)	7 (26.9)	8 (72.7)	2 (40.0)	7 (43.8)	7 (53.8)	0.112
Diffuse telangiectasia	13 (18.3)	4 (15.4)	2 (18.2)	2 (40.0)	4 (25.0)	1 (7.7)	0.546
Dots/glomerular vessel	14 (19.7)	3 (11.5)	2 (18.2)	0 (0.0)	6 (37.5)	3 (23.1)	0.181
Other							
Crusting	32 (45.1)	8 (30.8)^a^	2 (18.2)^a^	0 (0.0)^a^	14 (87.5)^b^	8 (61.5)^a,b^	< 0.001
Diffuse erythema	40 (56.3)	13 (50.0)^a,b^	2 (18.2)^a^	1 (20.0)^a,b^	13 (81.3)^b^	11 (84.6)^b^	0.001
Pustules	18 (25.4)	1 (3.8)^a^	0 (0.0)^a,b^	0 (0.0)^a,b^	12 (75.0)^c^	5 (38.5)^b,c^	< 0.001
Scales	59 (83.1)	22 (84.6)	10 (90.9)	3 (60.0)	13 (81.3)	11 (84.6)	0.708

*Note:* Comparisons were performed using the Pearson chi‐squared or Fisher's exact test, as appropriate. Post hoc pairwise comparisons were adjusted using the Bonferroni correction. Different superscript letters indicate significant differences after adjustment (*p* < 0.05).

Abbreviations: BD, black dot; DP, diffuse pustular type; GP, grey patch; K, kerion celsi; SD, seborrhoeic dermatitis‐like diffuse scaling.

#### 3.3.1 Follicular and Perifollicular Findings

Black dots (78.9%) and follicular hyperkeratosis (78.9%) were the most frequently observed follicular findings across all subtypes, without significant differences among clinical types. In contrast, empty follicles showed a significant difference by clinical subtype (*p* = 0.021), being particularly common in the seborrhoeic dermatitis‐like diffuse scaling type (72.7%) and the black dot type (60.0%), whereas relatively infrequent in the grey‐patch type (19.2%).

Perifollicular erythema and perifollicular pustules/vesicles were significantly associated with inflammatory variants. Perifollicular erythema was observed in 84.6% of the diffuse pustular type and 50.0% of kerion celsi cases, compared with 19.2% in the grey patch type (*p* = 0.002). Similarly, perifollicular pustules or vesicles were highly prevalent in inflammatory subtypes, particularly in kerion celsi (81.3%) and diffuse pustular type (92.3%), but were rare or absent in non‐inflammatory subtypes (*p* < 0.001). Perifollicular scales were common across all subtypes (76.1%) without significant differences.

#### 3.3.2 Hair Shaft Abnormalities

Among hair shaft abnormalities, broken hairs (78.9%) and bent hairs (64.8%) were the most frequently observed features. Comma hairs showed a significant association with clinical subtype (*p* = 0.008), being highly prevalent in the diffuse pustular (84.6%) and black dot types (80.0%), but markedly less frequent in the seborrhoeic dermatitis‐like diffuse scaling type (18.2%). Morse code–like hairs were most commonly seen in the grey‐patch type (69.2%), although this did not reach statistical significance (*p* = 0.139).

Bent hairs also occurred frequently across subtypes, with the highest prevalence in the grey‐patch (80.8%) and black dot types (60.0%). Broken hairs were consistently observed in over 70% of cases across all clinical classifications, without significant subtype‐specific differences (*p* = 0.586). Corkscrew hairs and zigzag hairs were present in approximately half of the cases (49.3% and 47.9%, respectively), with no statistically significant variation between subtypes.

#### 3.3.3 Vascular and Additional Scalp Features

Arborizing vessels were the most common vascular pattern (43.7%), with no significant differences among clinical subtypes. Other vascular morphologies, including dotted or glomerular vessels and diffuse telangiectasia, were less frequent and not subtype‐specific.

Additional scalp findings showed clear associations with inflammatory status. Crusting, diffuse erythema and pustules were significantly more frequent in inflammatory subtypes than in non‐inflammatory subtypes (*p* < 0.001, *p* = 0.001 and *p* < 0.001, respectively). Scaling was common across all subtypes (83.1%), with no significant differences.

### 3.4 Dermoscopic Features According to Causative Fungal Species

Dermoscopic findings stratified by fungal pathogens are summarized in Table 4 and Figure 1. Empty follicles were significantly more prevalent in *Trichophyton* infections (83.3%) than in *Microsporum* infections (29.4%) (*p* = 0.001). In contrast, several hair shaft abnormalities were significantly associated with *Microsporum* infections, including zigzag hairs (58.5% vs. 16.7%, *p* = 0.009), Morse code–like hairs (66.7% vs. 8.3%, *p* < 0.001) and bent hairs (74.5% vs. 41.7%, *p* = 0.040).

**TABLE 4:** Dermoscopic features of tinea capitis according to causative fungal pathogens (*n* = 63).

Dermoscopic feature	*Microsporum* (*n* = 51)	*Trichophyton* (*n* = 12)	*p*
Follicular opening			
Black dots	41 (80.4)	8 (66.7)	0.440
Fibrotic white dots	1 (2.0)	0 (0.0)	1.000
Follicular hyperkeratosis	38 (74.5)	11 (91.7)	0.270
Empty follicles	15 (29.4)	10 (83.3)	0.001
Perifollicular			
Perifollicular erythema	19 (37.3)	6 (50.0)	0.517
Perifollicular pustules/vesicles	21 (41.2)	4 (33.3)	0.749
Perifollicular scale	39 (76.5)	8 (66.7)	0.481
Hair pattern			
Broken hair	43 (84.4)	9 (75.0)	0.425
Comma hair	31 (60.8)	7 (58.3)	1.000
Corkscrew hair	28 (54.9)	5 (41.7)	0.409
Zigzag hair	30 (58.5)	2 (16.7)	0.009
Morse code–like hair	34 (66.7)	1 (8.3)	< 0.001
Bent hair	38 (74.5)	5 (41.7)	0.040
Vessels			
Arborizing vessels	21 (41.2)	7 (58.3)	0.282
Diffuse telangiectasia	10 (19.6)	2 (16.7)	1.000
Dots/glomerular vessel	12 (23.5)	1 (8.3)	0.431
Other			
Crusting	23 (45.1)	5 (41.7)	0.830
Diffuse erythema	29 (56.9)	5 (41.7)	0.342
Pustules	13 (25.5)	4 (33.3)	0.719
Scales	44 (86.3)	7 (58.3)	0.041

*Note:* Only cases with confirmed identification of *Microsporum* or *Trichophyton* were included. Comparisons between groups were conducted using the Pearson chi‐squared test or Fisher's exact test, as appropriate.

Broken hairs, comma hairs and corkscrew hairs were more frequently observed in *Microsporum* infections; however, these differences did not reach statistical significance. Among additional scalp features, scaling was significantly more common in *Microsporum* infections than in *Trichophyton* infections (86.3% vs. 58.3%, *p* = 0.041). No significant differences were observed in perifollicular inflammatory findings or vascular patterns between the two pathogen groups.

### 3.5 Super‐Resolution Confocal Microscopic Findings of Dystrophic Hairs

**FIGURE 2:**
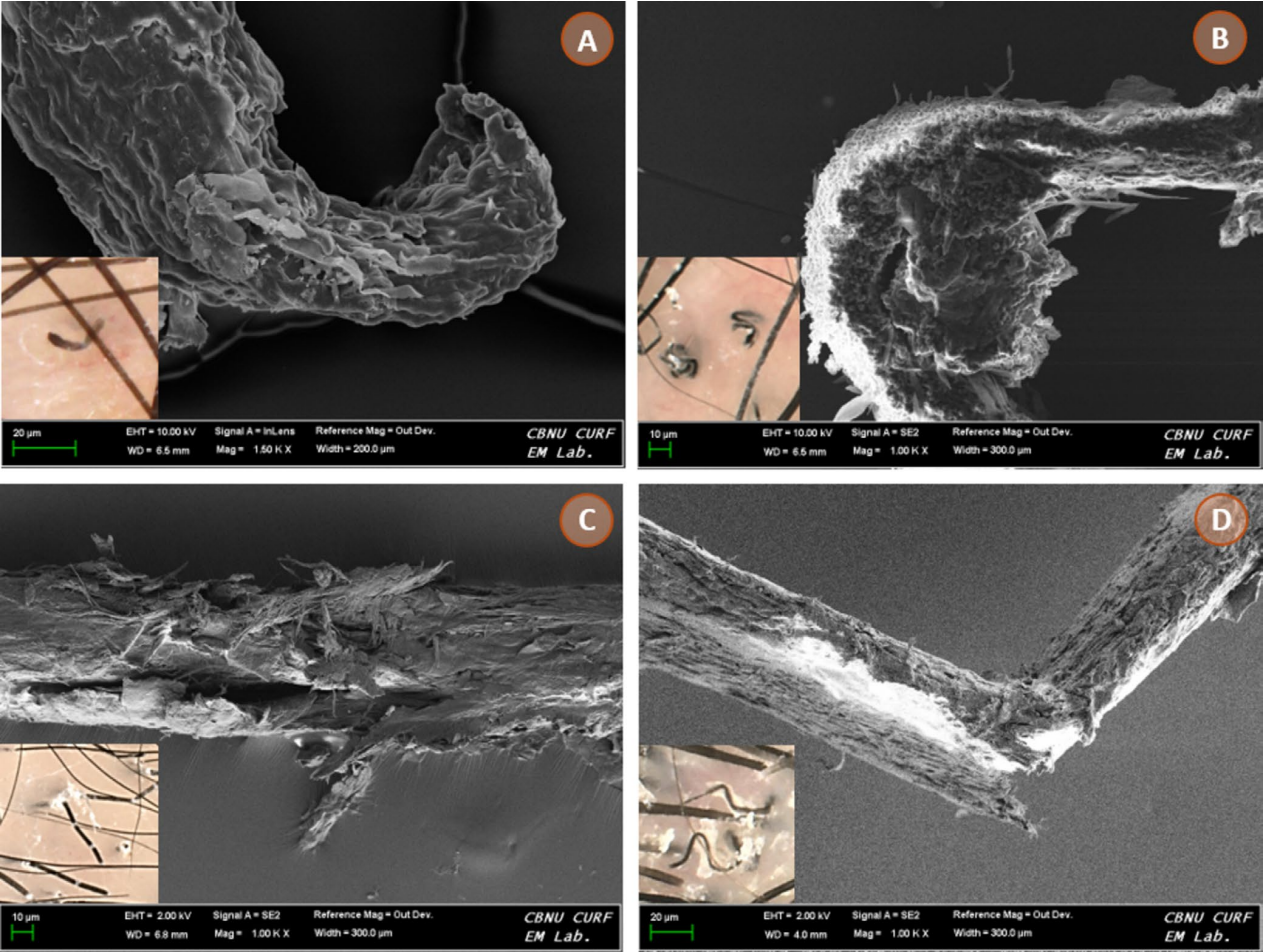
Super‐resolution confocal laser scanning microscopy (Airyscan) images demonstrating microscopic structural correlates of characteristic dermoscopic hair shaft abnormalities. (A) Comma hair and (B) corkscrew hair from a representative *Trichophyton* infection, showing dense fungal spores within the hair shaft and disruption of internal fibrillar structures. (C) Morse code–like hair and (D) zigzag hair associated with *Microsporum canis*, demonstrating incomplete transverse shaft fractures and focal weakened bent areas, consistent with structural compromise.

Super‐resolution confocal microscopy was performed on plucked hairs showing characteristic dermoscopic patterns in representative cases. In comma and corkscrew hairs, frequently observed in *Trichophyton* infections, the hair shafts were densely filled with fungal spores, with marked disruption and fragmentation of internal fibrillar bundles (Figure 2A,B). In contrast, hairs exhibiting Morse code–like and zigzag patterns, mainly associated with *Microsporum canis* infections, demonstrated multiple incomplete transverse shaft fractures and focal air‐filled gaps, resulting in weakened and bent areas along the shaft (Figure 2C,D). These findings corresponded to the horizontal white bands and angular deformities observed dermoscopically. Overall, microscopic analysis confirmed that distinct dermoscopic patterns reflect specific structural damage to the hair shaft caused by different modes of fungal invasion.

### 3.6 Dermoscopic Features According to Hair Curl Type

Dermoscopic findings, categorized by hair curl type, are presented in Table S1. Although certain features, such as empty follicles and perifollicular erythema, tended to be more frequent in patients with wavy or curly hair, no statistically significant associations were identified between hair curl type and any dermoscopic features, including hair shaft abnormalities, perifollicular changes, vascular patterns or additional scalp findings (all *p* > 0.05).

### 3.7 Dermoscopic Features of Age Groups Between Children and the Elderly

The distinct dermoscopic patterns of tinea capitis across two main age groups (≤ 10 and ≥ 61 years old) are shown in Table S2. Morse code–like hairs were significantly more prevalent in children (71.9% vs. 43.8%, *p* = 0.023). In contrast, elderly patients exhibited a significantly higher incidence of empty follicles (53.1% vs. 25.0%, *p* = 0.021) and perifollicular erythema (56.3% vs. 28.1%, *p* = 0.023). Other characteristics of the scalp, hair follicles, hair shape and blood vessels indicated no statistically significant difference between the two cohorts.

## 4 Discussion

In this single‐centre retrospective study, we comprehensively characterized dermoscopic features of TC in a Korean population and examined their associations with epidemiological characteristics, clinical subtypes, age groups and causative fungal species. By integrating detailed dermoscopic assessment with mycological confirmation, our study expands current understanding of the diagnostic and aetiological value of dermoscopy in TC, particularly in an adult‐predominant Asian cohort.

Although TC has traditionally been regarded as a childhood disease, our cohort demonstrated a distinct bimodal age distribution, with a substantial proportion of adults and elderly age groups. More than half of the patients were adults, and the elderly group (aged 61 years and above) accounted for a remarkably large proportion. This finding is consistent with recent epidemiological reports from Korea and other countries, which report an increasing incidence of adult‐onset TC [12, 13]. Proposed explanations include demographic ageing, immunosenescence, alterations in scalp sebum composition and close contact with domestic animals [14]. In line with this, zoonotic exposure was the most common suspected source of infection in our cohort, and *Microsporum canis* was the predominant pathogen, supporting the role of pet‐associated transmission in adult TC.

Hair curl type has been suggested to influence certain trichoscopic findings, particularly corkscrew hairs. However, we did not identify any statistically significant association between hair curl type and dermoscopic features, suggesting dermoscopic patterns of TC are primarily driven by fungal invasion patterns and host inflammatory responses rather than by intrinsic hair morphology.

Consistent with previous reports [15, 16, 17, 18, 19], black dots, follicular hyperkeratosis and scaling were the most frequent dermoscopic findings, reflecting their high sensitivity for TC. However, these signs lack specificity and may overlap with other alopecic disorders. In contrast, distinctive hair shaft abnormalities such as comma hairs, corkscrew hairs and Morse code–like hairs provided greater discriminatory value.

When analysed by clinical subtype, comma hairs were significantly associated with the diffuse pustular type, whereas Morse code–like hairs and bent hairs were more frequently observed in the grey patch type. Inflammatory variants, including kerion celsi and diffuse pustular type, showed prominent perifollicular erythema, pustules, vesicles, crusting and diffuse erythema. These inflammatory changes may obscure characteristic hair shaft abnormalities, which could explain why specific dermoscopic markers are sometimes less apparent in severe inflammatory TC.

An important finding of our study is the significant association between dermoscopic features and causative fungal species. Morse code–like hairs, zigzag hairs and bent hairs were significantly more prevalent in *Microsporum* infections, whereas empty follicles were strongly associated with *Trichophyton* infections. These observations are biologically plausible and align with established invasion patterns: *Microsporum* species typically exhibit ectothrix parasitism, leading to focal structural weakening and horizontal white bands, whereas *Trichophyton* species more commonly show endothrix invasion, which may result in complete shaft destruction and empty follicles [20].

These dermoscopic‐mycological correlations were further supported by ultrastructural analysis of plucked diseased hairs using super‐resolution confocal laser scanning microscopy. Comma and corkscrew hairs demonstrated dense fungal spores filling the shaft, while Morse code–like and zigzag hairs showed incomplete transverse fractures and weakened bent areas, corresponding to structural compromise caused by fungal invasion. Notably, however, certain short broken hair patterns, such as comma and corkscrew hairs, were also observed in *Microsporum* infections. Extensive shaft fragility in severe cases may similarly result in completely fractured short hairs, suggesting that these findings should not be regarded as strictly species‐specific markers. Instead, dermoscopic features in tinea capitis may represent species‐ and severity‐dependent markers, reflecting not only the underlying fungal invasion pattern but also the extent of inflammatory burden and hair shaft fragility.

Therefore, although dermoscopy cannot replace mycological confirmation, the presence of characteristic findings such as Morse code–like hairs may provide early aetiological clues and support timely diagnostic suspicion while awaiting laboratory results, rather than directly determining antifungal selection [19]. In addition, dermoscopy plays an important role in differentiating tinea capitis from other causes of patchy alopecia, such as alopecia areata and trichotillomania. Although black dots and broken hairs may overlap, the coexistence of diffuse scaling with fungal‐specific hair shaft abnormalities strongly favours tinea capitis. Alopecia areata typically shows exclamation‐mark hairs, whereas trichotillomania is characterized by hairs broken at varying lengths without prominent scaling [15, 16, 18, 21]. These findings emphasize the importance of pattern recognition rather than reliance on single dermoscopic signs.

From a clinical perspective, these implications are particularly relevant in adult‐predominant or *Microsporum*‐endemic settings, where delayed diagnosis and misclassification as seborrhoeic dermatitis are common. This challenge is especially evident in the seborrhoeic dermatitis–like diffuse scaling subtype, in which highly specific indicators such as comma or corkscrew hairs occur less frequently. Therefore, careful recognition of subtle findings, including focal black dots or broken hairs within diffuse scaling, may help prevent inappropriate steroid use and delayed treatment.

This study has several limitations. Its retrospective design and single‐centre setting may limit generalizability, particularly in regions where *Trichophyton tonsurans* predominates. The predominance of *Microsporum canis* reflects local epidemiology and may influence the distribution of dermoscopic features. In addition, interobserver variability was not formally assessed, and multivariate analyses were limited by sample size. Despite these limitations, our study provides the first comprehensive analysis of dermoscopic features of TC in a Korean population, integrating epidemiological, clinical and mycological perspectives. Future prospective multicentre studies with larger sample sizes are warranted to validate the predictive value of specific dermoscopic signs and to develop diagnostic algorithms applicable across diverse populations.

In conclusion, dermoscopy is a valuable adjunctive tool for diagnosing and characterizing TC. By demonstrating subtype‐ and pathogen‐specific dermoscopic patterns and correlating them with hair shaft damage, our study provides novel evidence from an underrepresented population and supports the integration of dermoscopy into routine clinical practice.

## Author Contributions

**Jin Park:** conceptualization, funding acquisition, resources, software, supervision, writing – original draft, writing – review and editing. **Hyun‐Ji Ryu:** investigation. **Gwangil Kim:** software, visualization. **Seok‐Kweon Yun:** supervision, writing – review and editing. **Thi Quynh Trang Tran:** data curation, formal analysis, investigation, validation, writing – original draft. **Su‐Kyung Park:** investigation, methodology. **Kyung‐Hwa Nam:** project administration, validation, writing – review and editing. **HyoHyun Yoo:** investigation, validation.

## Funding

This study was supported by the Fund of Biomedical Research Institute of Jeonbuk National University Hospital, Korea Health Technology R&D Project through the Korea Health Industry Development Institute (KHIDI) by the Ministry of Health and Welfare of the Republic of Korea (RS‐2023‐KH136575) and the Bio&Medical Technology Development Program of the National Research Foundation (NRF) funded by the Korean government (MSIT) (No. RS‐2023‐00236157).

## Conflicts of Interest

The authors declare no conflicts of interest.

## Supporting information


**Table S1:** summarizes the dermoscopic features of tinea capitis according to hair curl type, including straight, wavy and curly hair.
**Table S2:** presents a subgroup analysis comparing dermoscopic findings between patients aged ≤ 10 years and those aged ≥ 61 years, showing age‐related differences in empty follicles, perifollicular erythema and Morse code–like hair.

## Data Availability

The data supporting the findings of this study are available upon request from the corresponding author. The data are not publicly available due to privacy or ethical restrictions.
