# Supplementary material for: Dermoscopic Features of Tinea Capitis in Korean Patients: Correlation With Clinical Subtypes, Age Groups and Causative Dermatophytes
Source: Mycoses. 2026 May 18;69:e70184. doi: 10.1111/myc.70184 (PMC13182684; doi:10.1111/myc.70184)
Supplement: Supplementary file 1 — Table S1: summarizes the dermoscopic features of tinea capitis according to hair curl type, including straight, wavy and curly hair. Table S2: presents a subgroup analysis comparing dermoscopic findings between patients aged ≤ 10 years and those aged ≥ 61 years, showing age‐related differences in empty follicles, perifollicular erythema and Morse code–like hair. [file MYC-69-e70184-s001.docx]

**Supplementary Table S1.** **Dermoscopic features of tinea capitis according to hair curl type (n=71)**

| Dermoscopic feature | Straight  (n=45) | Wavy  (n=19) | Curly  (n=7) | p-value |
| --- | --- | --- | --- | --- |
| **Follicular opening** |  |  |  |  |
| Black dots | 34 (75.6) | 15 (78.9) | 7 (100.0) | 0.165 |
| Fibrotic white dots | 1 (2.2) | 0 (0.0) | 0 (0.0) | 0.631 |
| Follicular hyperkeratosis | 33 (73.3) | 16 (84.2) | 7 (100.0) | 0.108 |
| Empty follicles | 15 (33.3) | 12 (63.2) | 3 (42.9) | 0.088 |
| **Perifollicular** |  |  |  |  |
| Perifollicular erythema | 13 (33.3) | 12 (63.2) | 4 (57.1) | 0.066 |
| Perifollicular pustules/vesicles | 16 (35.6) | 11 (57.9) | 3 (42.9) | 0.257 |
| Perifollicular scale | 32 (71.1) | 16 (84.2) | 6 (85.7) | 0.437 |
| **Hair pattern** |  |  |  |  |
| Broken hair | 35 (77.8) | 14 (73.7) | 7 (100.0) | 0.162 |
| Comma hair | 25 (55.6) | 12 (63.2) | 5 (71.4) | 0.663 |
| Corkscrew hair | 22 (48.9) | 8 (42.1) | 5 (71.4) | 0.404 |
| Zigzag hair | 22 (48.9) | 8 (42.1) | 4 (57.1) | 0.773 |
| Morse code-like hair | 24 (53.3) | 9 (47.4) | 5 (71.4) | 0.541 |
| Bent hair | 31 (68.9) | 10 (52.6) | 5 (71.4) | 0.436 |
| **Vessels** |  |  |  |  |
| Arborizing vessels | 16 (35.6) | 10 (52.6) | 5 (71.4) | 0.132 |
| Diffuse telangiectasia | 7 (15.6) | 3 (15.8) | 3 (42.9) | 0.277 |
| Dots/glomerular vessel | 10 (22.2) | 3 (15.8) | 1 (14.3) | 0.775 |
| **Other** |  |  |  |  |
| Crusts | 21 (46.7) | 8 (42.1) | 3 (42.9) | 0.938 |
| Diffuse erythema | 24 (53.3) | 13 (68.4) | 3 (42.9) | 0.398 |
| Pustules | 11 (24.4) | 6 (31.6) | 1 (14.3) | 0.637 |
| Scales | 37 (82.2) | 15 (78.9) | 7 (100.0) | 0.242 |

Comparisons were performed using the Pearson chi-square test or Fisher’s exact test, as appropriate.

**Supplementary Table S2.** **Dermoscopic features of tinea capitis according to age group (n=64)**

| Dermoscopic feature | ≤ 10 years  (n=32) | ≥ 61 years (n=32) | p-value |
| --- | --- | --- | --- |
| **Follicular opening** |  |  |  |
| Black dots | 26 (81.3) | 23 (71.9) | 0.376 |
| Fibrotic white dots | 1 (3.1) | 0 (0.0) | 1.000 |
| Follicular hyperkeratosis | 25 (78.1) | 26 (81.3) | 0.756 |
| Empty follicles | **8 (25.0)** | **17 (53.1)** | **0.021** |
| **Perifollicular** |  |  |  |
| Perifollicular erythema | **9 (28.1)** | **18 (56.3)** | **0.023** |
| Perifollicular pustules/vesicles | 11(34.4) | 16 (50.0) | 0.206 |
| Perifollicular scale | 25 (78.1) | 25 (78.1) | 1.000 |
| **Hair pattern** |  |  |  |
| Broken hair | 26 (81.3) | 23 (71.9) | 0.376 |
| Comma hair | 19 (59.4) | 18 (56.3) | 0.800 |
| Corkscrew hair | 13 (40.6) | 19 (59.4) | 0.134 |
| Zigzag hair | 20 (62.5) | 13 (40.6) | 0.080 |
| Morse code-like hair | **23 (71.9)** | **14 (43.8)** | **0.023** |
| Bent hair | 24 (75.0) | 18 (56.3) | 0.114 |
| **Vessels** |  |  |  |
| Arborizing vessels | 11 (34.4) | 18 (56.3) | 0.079 |
| Diffuse telangiectasia | 6 (18.8) | 5 (15.6) | 0.740 |
| Dots/glomerular vessel | 9 (28.1) | 4 (12.5) | 0.120 |
| **Other** |  |  |  |
| Crusts | 17 (53.1) | 13 (40.6) | 0.316 |
| Diffuse erythema | 20 (62.5) | 18 (56.3) | 0.611 |
| Pustules | 8 (25.0) | 8 (25.0) | 1.000 |
| Scales | 27 (84.4) | 27 (84.4) | 1.000 |

Only patients aged ≤10 or ≥61 years were included in this subgroup analysis (n = 64). Comparisons were performed using the Pearson chi-square test or the Fisher’s exact test, as appropriate.
